# Fracture prevalence and its association with bone density among children living with HIV in Zimbabwe

**DOI:** 10.1097/QAD.0000000000003477

**Published:** 2023-02-07

**Authors:** Ruramayi Rukuni, Victoria Simms, Andrea M. Rehman, Cynthia Mukwasi-Kahari, Hilda Mujuru, Rashida A. Ferrand, Celia L. Gregson

**Affiliations:** a Clinical Research Department, Faculty of Infectious and Tropical Diseases, London School of Hygiene & Tropical Medicine, London, UK; b The Health Research Unit Zimbabwe, Biomedical Research and Training Institute, Harare, Zimbabwe; c MRC International Statistics and Epidemiology Group, Faculty of Epidemiology and Population Health; d Department of Infectious Disease Epidemiology, Faculty of Epidemiology and Population Health, London School of Hygiene & Tropical Medicine, London, UK; e Faculty of Medicine and Health Sciences, University of Zimbabwe, Harare, Zimbabwe; f Musculoskeletal Research Unit, Translational Health Sciences, Bristol Medical School, University of Bristol, Bristol, UK.

**Keywords:** fracture, bone, HIV, paediatric, disability

## Abstract

HIV infection impairs bone density in children living with HIV (CLWH). We aimed to determine the prevalence of self-reported fracture (past or current), associated risk factors and disability, by HIV status in Zimbabwean children.

**Design:**

Cross-sectional study.

**Methods:**

We recruited CLWH aged 8–16 years taking antiretroviral therapy (ART) for ≥2 years from HIV clinics, and HIV-uninfected children from schools in Harare. Interviewer-administered questionnaires collected data on fracture site and management, sociodemographics, dietary calcium and vitamin D, physical activity and HIV history. Dual-energy X-ray absorptiometry (DXA) measured size-adjusted bone density.

**Results:**

We recruited 303 CLWH [mean (SD) age 12.5 (2.5) years; 50% female] and 306 children without HIV [12.5 (2.5) years; 51% female]. Median age at HIV diagnosis in CLWH was 3.0 years [interquartile range (IQR) 1.2, 5.9], and median ART duration 8.1 years [IQR 6.2, 9.5]. 53.8% CLWH had self-reported disability and/or functional impairment, vs. 29.4% children without HIV. Fracture prevalence was 5.9% with no difference by HIV status [21/306 (6.9%) vs. 14/303 (4.6%), *P* = 0.24]. Male sex was associated with fractures. Low size-adjusted bone density (*Z*-score < −2) was associated with prevalent fractures in CLWH {risk ratio [RR] 1.14 (95% confidence interval (CI) −0.02, 2.29]}, but not in children without HIV [RR −0.04 (−2.00, 1.91)], *P*-interaction = 0.27. All sought medical attention for their fracture(s), but CLWH were less often admitted to hospital [2/14 (14.3%) vs. 7/21 (33.3%)].

**Conclusion:**

Prevalent fractures may be associated with low lumbar spine bone density in CLWH. Fracture surveillance and strategies to reduce future fracture risk are warranted as CLWH enter adulthood.

## Introduction

Fractures are relatively common in healthy children, probably related to high levels of physical activity during childhood [1]. In one British study, around one-third of all children experienced at least one fracture before the age of 17 years [2]. A study in South Africa reported fracture incidence rates of 12–38 per 1000 children per year with higher rates in boys above the age of 10 years, likely due to greater physical risk-prone activities [3]. Childhood fractures impact daily activities, with upper limb fractures (the most common fracture site) leading to an average of 14 activity restricted days per child in high-income countries; similarly lower limb fractures are responsible for on average 26 activity restricted days [4].

As well as activities that may put individuals at risk of injury, impairments in bone architecture and quality may increase fracture risk. In high-income settings, a number of prospective studies have demonstrated an association between low bone mass and childhood fracture risk [1], for example in a UK community-based cohort study, a one standard deviation (SD) reduction in bone mass was associated with an 89% [odds ratio (OR) 1.89, 95% confidence interval (CI) 1.18, 3.04, *P* = 0.009] increased fracture risk [5].

In sub-Saharan Africa (SSA), up to 50% of children with perinatally acquired HIV infection experience stunting [6], and we have shown evidence of marked deficits in size-adjusted bone-density among peri-pubertal children living with HIV (CLWH) in Zimbabwe [7]. It is currently unclear whether the low bone mass seen in CLWH [7–9], confers an increase in fracture risk. Recent data from the United States suggest fracture incidence is higher in CLWH, but only in those younger than 6 years [10]. Certainly, fracture rates are higher in adults living with HIV compared to the general population [11,12]. Hence, this study aimed to determine the prevalence of fractures in CLWH, to explore the relationship between bone density and fracture prevalence, and to understand the extent to which fractures are associated with functional impairment and disabilities.

## Materials and methods

### Study design and participants

A cross-sectional study, using baseline data from the IMVASK study (The IMpact of Vertical HIV infection on child and Adolescent SKeletal development in Harare, Zimbabwe), was conducted as per published protocol (ISRCTN12266984) [13]. The bone density findings have been published previously [7]. CLWH, aged 8–16 years, were recruited from outpatient HIV clinics at the two large public-sector general hospitals in Harare (Sally Mugabe and Parirenyatwa Hospitals). Paediatric studies suggest antiretroviral therapy (ART) initiation prompts an initial decline in bone mass which stabilizes after two years [14], hence we enrolled CLWH who had been taking ART for at least 2 years. Systematic quota-based sampling, stratified by age and sex, was used to recruit 50 male and 50 female CLWH in each of three age-groups (8–10, 11–13 and 14–16 years). Exclusion criteria included being acutely unwell (defined as requiring immediate hospitalization), not residing in Harare and children being unaware of their HIV status (to avoid inadvertent disclosure through study participation). A maximum of five CLWH were recruited each day for logistical reasons.

For comparison, children without HIV were recruited from six government primary and secondary schools randomly selected from the 109 primary and 44 secondary schools within the same catchment areas in Harare served by the hospitals. Younger children (8–12 years) were sampled from primary schools and older children (14–16 years) from secondary schools, with 13-year-olds sampled from both school types. The number of children selected from each school was proportional to school size, giving each child equal probability of being sampled. A random number sequence applied to school registers was employed to select participants using the same quota-based sampling approach of 50 males and 50 females in each of the three age strata (8–10, 11–13 and 14–16 years). Children underwent HIV testing before recruitment, and those testing positive and not in care were referred to HIV services.

### Study questionnaire

An interviewer-administered questionnaire was used to collect sociodemographic data and information regarding self-recalled fractures sustained since birth, from children and caregivers. Details of the fracture site and clinical management were recorded. The International Physical Activity Questionnaire (IPAQ), validated in multiple countries including South Africa, assessed physical activity as multiples of the resting metabolic rate (MET) in MET-minutes [15]. Diet and nutrition were assessed using a tool based on a validated dietary diversity and food frequency tool from India and Malawi, adapted to the Zimbabwean context, using international guidelines applicable to SSA [16]. This tool quantified dietary calcium and vitamin D intake, plus sunlight exposure; adaptations reflected the local context where fortification of oils and margarine with vitamin D is mandated and specific calcium and vitamin D rich foods, for example, kapenta fish, are commonly eaten.

### Anthropometry

Anthropometric measurements were carried out by trained research staff. Standing and sitting height, measured to the nearest 0.1 cm (using a Seca 213 stadiometer), and weight (using Seca 875 weight scales), measured to the nearest 0.1 kg, were taken by two separate readers. If height measurements differed by more than 0.5 cm, or weight measurements by more than 0.5 kg, a third reading was taken, and final height and weight values were taken as means of the two or three measurements. The same researchers measured both the children with and without HIV. All equipment was calibrated annually.

### HIV assessment

Details about HIV including age at HIV diagnosis, ART regimen and duration were collected from hand-held medical records during the interviewer-administered questionnaire for CLWH. CD4^+^ cell count was measured using an Alere PIMA CD4 machine (Waltham, Massachusetts, USA) and HIV viral load using the GeneXpert HIV-1 viral load platform (Cepheid Inc., Sunnyvale, California, USA), with viral suppression defined as <1000 copies/ml as per WHO guidelines [17].

### Dual energy X-ray absorptiometry scanning

Dual-energy X-ray absorptiometry (DXA) scans of the lumbar spine and total body were performed by one of two trained radiographers using standard procedures on a Hologic QDR Wi densitometer (Hologic Inc., Bedford, Massachusetts, USA) with Apex Version 4.5 software for scan analysis. The manufacturer provided spine phantom was used daily for calibration. DXA scans were repeated in a subgroup (*n* = 30) to determine reproducibility. The precision error was a root mean square-standard deviation (RMS-SD) of 0.011 g/cm^2^ (lumbar spine) and 0.010 g/cm^2^ (total body) with an RMS-coefficient of variation (CV) of 1.35% (lumbar spine) and 1.22% (total body). An important limitation of DXA in paediatric populations with chronic disease is that the two-dimensional (areal) bone density values are highly dependent upon body, and therefore bone size; hence DXA underestimates bone density in small children [18]. The two main size adjustment techniques recommended by the International Society for Clinical Densitometry (ISCD) to overcome the problem of size dependence of DXA measurement [19], were employed, that is to measure total-body less-head (TBLH) bone mineral content (BMC) for lean mass adjusted for height (TBLH-BMC^LBM^) and lumbar spine bone mineral apparent density (low size-adjusted bone density [LS-BMAD]). LS-BMAD was calculated from DXA-measured lumbar spine data using the Carter method [20]. TBLH-BMC^LBM^ was calculated from the whole-body scan using published derived equations, for Hologic DXAs, which adjust for log-transformed total body lean mass, total body fat mass and height [21]. Sex and age-matched *Z*-scores were generated using Hologic UK population reference data as recommended by ISCD guidelines, as there were no local reference data available [19]. Low TBLH-BMC^LBM^ and LS-BMAD were defined as *Z*-score <−2.0 [21].

### Statistical analysis

A sample size of 300 in each group was calculated to enable detection of a difference between children with and without HIV in fracture prevalence of 7% (predicted 5% in children without HIV and 12% in CLWH) with 80% power and a significance level of 0.05.

Height-for-age and weight-for-age *Z*-scores were calculated using 1990 UK reference data [7], with *Z*-scores <−2.0 defining stunting and underweight respectively. Socio-economic status (SES) was derived using the first component from a principal component analysis combining an asset list (detailing: number in household, home ownership, access to electricity, water, a flush toilet and/or pit latrine and ownership of a bicycle, car, fridge, television, and/or radio), which was categorised into tertiles for analysis.

Analyses were conducted using Stata 17.0 (StatCorp, College Station, Texas, USA). The primary exposure was HIV and primary outcome the prevalence of self-reported fracture. The secondary outcome was the prevalence of disability in those with self-reported fracture. The characteristics of participants with HIV were compared with those without HIV, using independent sample t-tests for means, with unequal variance as required and chi-squared or Fisher's exact tests for proportions. Generalized linear log-binomial modelling was used to determine potential risk factors associated with prevalent fracture, including age [1], male [1], height [22], underweight [23], more physically active [24], living with HIV [25], reporting low calcium [26] and vitamin D intake [27], socio-economic deprivation indicated by low SES [28] and/or orphanhood [7]. A generalized linear log-binomial model was used to assess the association between size-adjusted bone density and prevalent fracture adjusting for age and sex.

### Ethical considerations

Ethical approvals were granted by the Medical Research Council of Zimbabwe (Ref: MRCZ/A/2297), the Institutional Review Board of the Biomedical Research and Training Institute in Harare (Ref: AP145/2018), the Joint Research Ethics Committee for University of Zimbabwe Faculty of Medicine and Health Sciences and the Parirenyatwa Group of Hospitals (Ref: 11/18), Sally Mugabe (formally Harare Central) Hospital Ethics Committee (Ref: 170118/04), and the London School of Hygiene & Tropical Medicine Ethics Committee (Ref: 15333). Parents/guardians provided written informed consent for study participation and HIV testing, and children provided written assent.

## Results

### Study population

We recruited 303 children living with HIV (CLWH) [mean (SD) age 12.5 (2.5) years; 50% female] and 306 children without HIV [mean (SD) age 12.5 (2.5) years; 51% female]. As previously reported, CLWH were more likely to be in the lowest socioeconomic tertile, orphaned (one or both parents dead) and to report lower levels of physical activity compared with children without HIV [7]. Reported dietary calcium and vitamin D intakes were similar between CLWH and children without HIV. Amongst CLWH, the median age at HIV diagnosis was 3.0 years [interquartile range (IQR) 1.2, 5.9], with median ART duration being 8.1 years [IQR 6.2, 9.5]; in 34% of cases, ART included tenofovir (Table 1).

**Table 1:** Socio-demographic, lifestyle and HIV characteristics of study participants.

		HIV positive		HIV negative		*P*-value
		*N*	*n* (%)^a^	*N*	*n* (%)^a^	
*Socio-demographic factors*	Age years, mean (SD)	303	12.5 (2.5)	306	12.5 (2.5)	0.793
	Female sex	303	151 (49.8)	306	155 (50.7)	0.840
	Socioeconomic status (SES)	303		306		0.005
	Tertile 1 (low)		115 (38.0)		88 (28.8)	
	Tertile 2 (middle)		105 (34.7)		98 (32.0)	
	Tertile 3 (high)		83 (27.4)		120 (39.2)	
	Orphaned (one or both parents dead)	297^b^	130 (43.8)	303	20 (6.6)	<0.001
	In school	303	299 (98.7)	306	306 (100)	0.06
*Lifestyle factors*	Physical activity level	303		306		0.012
	Low, <600 MET min/week		148 (48.8)		114 (37.3)	
	Moderate, 600–3000 MET min/week		77 (25.4)		88 (28.8)	
	High, >3000 MET min/week		78 (25.7)		104 (34.0)	
	Daily dietary calcium intake	303		306		0.936
	Very low, <150 mg/day		135 (44.6)		136 (44.4)	
	Low, 150–299 mg/day		62 (20.5)		66 (21.6)	
	Moderate, 300–450 mg/day		106 (35.0)		104 (34.0)	
	Daily dietary vitamin D	303		306		0.413
	Very low, <4.0 mcg/day		17 (5.6)		15 (4.9)	
	Low, 4.0–5.9 mcg/day		181 (59.7)		164 (53.6)	
	Moderate, 6.0–10.0 mcg/day		105 (34.7)		127 (41.5)	
*HIV characteristics*	Age at HIV diagnosis years, median (IQR)	303	3.0 (1.2–5.9)			
	Age at ART initiation years, median (IQR)	303	3.7 (1.8–6.9)			
	ART duration years, median (IQR)	303	8.1 (6.2–9.5)			
	% life on ART, mean (SD)	303	65.3 (22.0)			
	Current tenofovir use	303	102 (33.7)			
	Viral load <1000 copies/ml	268^c^	212 (79.1)			
	CD4^+^ cell count <500 cells/μl	288^d^	58 (20.1)			
*Anthropometry*	Height-for-age *Z*-score, mean (SD)	301^e^	−1.65 (1.12)	306	−0.59 (1.02)	<0.001
	Height-for-age *Z*-score <−2	301	95 (31.6)	306	22 (7.2)	<0.001
	Weight-for-age *Z*-score, mean (SD)	303	-1.47 (1.19)	305^f^	-0.50 (1.09)	<0.001
	Weight-for-age *Z*-score <−2	303	80 (26.4)	305	23 (7.5)	<0.001

a *n* (%) unless stated otherwise.

b Orphan status data was missing for nine participants (6 living with HIV, 3 HIV negative). the child's mother was alive but it was not known whether the father was still alive.

c Viral load data was missing in 35 participants with HIV.

d CD4^+^ data was missing in 15 participants; not fasted so bloods not taken (*n* = 12), refusal (*n* = 3).

e Missing height data for two participants with HIV.

f Missing weight data in one child without HIV.

### Prevalent fracture by HIV status

**Fig. 1:**
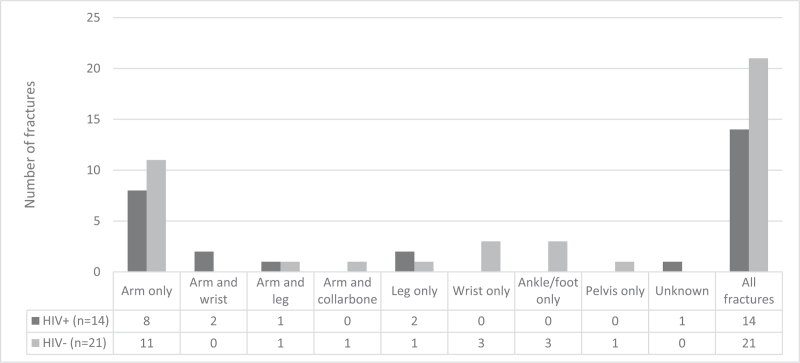
Prevalent fractures by skeletal site and HIV status.

Overall, 5.7% of children reported having experienced a fracture, with no difference in prevalence by HIV status [14 out of 303 (4.6%) in CLWH vs. 21 out of 306 (6.9%) in children without HIV; *P* = 0.235]. The most common fracture site was the arm and/or wrist in both CLWH and those without HIV [10/14 (71.4%) vs. 14/21 (66.7%)] (Fig. 1). Most fractures [31/35 (88.6%)] were confirmed by X-ray and in all cases, medical attention was sought to manage the fracture. Compared to children without HIV, CLWH were less likely to be admitted to hospital with their fracture [2/14 (14.3%) vs. 7/21 (33.3%)] (Figure 1, Supplemental Digital Content). No child had their fracture managed surgically.

### Self-reported disability

CLWH were more likely than children without HIV to self-report disability and/or functional impairment [163/303 (53.8%) vs. 90/306 (29.4%), chi^2^ = 37.3, *P* < 0.001]. CLWH were more likely to have difficulty walking and/or running [25/303 (8.3%) vs. 9/306 (2.9%), chi^2^ = 8.14, *P* = 0.004] compared to children without HIV. Difficulties with self-care were few [6/303 (2.0%) vs. 3/306 (1%) in those with and without HIV respectively, *P* = 0.34], whilst self-reported sensory, memory and speech difficulties were over twice as common in CLWH ([142/303 (46.9%) vs. 61/306 (19.9%), chi^2^ = 49.7, *P* < 0.001).

There was no clear evidence that prevalent fracture was associated with all-cause disability or with difficulty walking/running, either in CLWH or children without HIV, or overall (Table 2).

**Table 2:** Relationship between reported disability and prevalent fractures.

	*n*	Prevalent fracture, *n* (%)	Chi square	*P*-value
All children				
Self-reported disability	253	11 (4.4)	1.56	0.21
No disability	356	24 (6.7)		
Children with HIV				
Self-reported disability	163	4 (2.5)	3.76	0.060^a^
No disability	140	10 (7.1)		
Children without HIV				
Self-reported disability	90	7 (7.8)	0.17	0.68
No disability	216	14 (6.5)		
All children				
Self-reported difficulty walking/running	34	4 (11.8)	2.41	0.12^a^
No difficulty	575	31 (5.4)		
Children with HIV				
Self-reported difficulty walking/running	25	3 (12.0)	3.37	0.098^1^
No difficulty	278	11 (4.0)		
Children without HIV				
Self-reported difficulty walking/running	9	1 (11.1)	0.26	0.48
No difficulty	297	20 (6.7)		

a Fisher's exact test.

### Bone density and prevalent fracture

When examined overall, no clear association between low LS-BMAD or TBLH-BMC^LBM^*Z*-score with self-reported prevalent fracture was observed. However, when stratified by HIV status, there was weak evidence that CLWH with a low LS-BMAD *Z*-score had increased risk of self-reported fracture compared to CLWH with normal LS-BMAD (RR 1.14 [−0.02, 2.29], *P* = 0.053; this association was not seen among children without HIV, *P*-interaction = 0.27. Only two CLWH reported a prevalent fracture and had a low TBLH-BMC^LBM^*Z*-score (Table 3).

**Table 3:** The association between size-adjusted bone density and self-reported prevalent fracture in children living with and without HIV.

	DXA *Z*-score <−2	DXA *Z*-score ≥−2	RR (95% CI)	*P*-value
^a^LS-BMAD (*n* = 576)				
All children	5/56 (8.9)	25/520 (4.8)	0.62 (−0.31, 1.54)	0.187
Children with HIV	4/39 (10.3)	8/243 (3.3)	1.14 (−0.02, 2.29)	0.053
Children without HIV	1/17 (5.9)	17/277 (6.1)	−0.04 (−2.00, 1.91)	0.966
^b^TBLH-BMC^LBM^ (*n* = 575)				
All children	2/45 (4.4)	28/530 (5.3)	−0.17 (−1.57, 1.23)	0.809
Children with HIV	2/28 (7.1)	10/254 (3.9)	0.60 (−0.87, 2.06)	0.426
Children without HIV	0/17 (0.0)	18/276 (6.5)	-	-

DXA, dual-energy X-ray absorptiometry; LS-BMAD, low size-adjusted bone density; TBLH-BMC, total-body less-head bone mineral content.

a LS BMAD *Z*-score data is missing for 21 participants with HIV and 12 participants without HIV.

b TBLH-BMC^LBM^*Z*-score data is missing for 21 participants with HIV and 13 participants without HIV.

### Risk factors for prevalent fracture

Generalized linear log-binomial modelling identified male sex as a risk factor for reporting a prevalent fracture (Table 4). HIV status, SES, orphan status, physical activity, dietary vitamin D and calcium intake, height and weight were not associated with self-reported prevalent fracture after adjusting for age and sex (Table 4). Amongst those with HIV, age of HIV diagnosis, age at ART initiation, ART duration, tenofovir use, viral load and CD4^+^ cell count were not associated with self-reported prevalent fracture (Table 1, Supplemental Digital Content).

**Table 4:** Risk factors for reporting a prevalent fracture in all participants.

Risk factor	Prevalent fracture (*n* = 35) *N* (%)	No prevalent fracture (*n* = 574) *N* (%)	Age/sex adjusted RR (95% CI)	*P*-value
Age				
<12 years	12 (4.5)	255 (95.5)	Ref	0.228
≥12 years	23 (6.7)	319 (93.3)	0.42 (−0.26, 1.09)^a^	
Sex				
Male	23 (7.6)	280 (92.4)	Ref	0.054
Female	12 (3.9)	294 (96.1)	−0.67 (−1.35, 0.01)^b^	
Socioeconomic status (SES)				
Tertile 1 (low)	10 (4.9)	193 (95.1)	Ref	0.268
Tertile 2 (middle)	9 (4.4)	194 (95.6)	−0.14 (−1.02, 0.74)	
Tertile 3 (high)	16 (7.9)	187 (92.1)	0.45 (−0.31, 1.22)	
Orphan status (one or both parents dead)				
Yes	6 (4.0)	144 (96.0)	−0.57 (−1.43, 0.29)	0.196
No	29 (6.4)	421 (93.6)	Ref	
Missing	0 (0.0)	9 (100.0)		
Physical activity				
Low, <600 MET min/week	15 (5.7)	247 (94.3)	Ref	0.961
Moderate, 600–3000 MET min/week	9 (5.5)	156 (94.5)	−0.09 (−0.89, 0.71)	
High, >3000 MET min/week	11 (6.0)	171 (94.0)	0.02 (−0.73, 0.78)	
Dietary calcium intake				
<150 mg/day	15 (5.5)	256 (94.5)	−0.03 (−0.77, 0.70)	0.944
150–299 mg/day	8 (6.3)	120 (93.8)	0.11 (−0.75, 0.97)	
300–449 mg/day	12 (5.7)	198 (94.3)	Ref	
Dietary vitamin D intake				
<6.0 mcg/day	20 (5.3)	357 (94.7)	−0.17 (−1.81, 0.48)	0.616
≥6.0 mcg/day	15 (6.5)	217 (93.5)	Ref	
Height for age *Z*-score				
Stunted (<−2)	4 (3.4)	113 (96.6)	−0.72 (−1.74, 0.30)	0.167
Not stunted	31 (6.3)	459 (93.7)	Ref	
Missing	0 (0.0)	2 (100.0)		
Weight-for-age *Z*-score				
Underweight (<−2)	8 (7.8)	95 (92.2)	0.22 (−0.55, 0.99)	0.583
Not underweight	27 (5.4)	478 (94.6)	Ref	
Missing	0 (0.0)	1 (100.0)		
HIV status				
Positive	14 (4.6)	289 (95.4)	−4.26 (−1.08, 0.23)	0.203
Negative	21 (6.9)	285 (93.1)	Ref	

CI, confidence interval; RR, risk ratio.

a RR adjusted for sex only.

b RR adjusted for age only.

## Discussion

Our study found a reported fracture prevalence of 5.9% among peripubertal children. Observed fracture patterns were similar to previous reports; upper limb fractures accounting for 65% of fractures [29] and lower limb up to 28% [30]. Fracture risk is explained by both extrinsic activities that put individuals at risk of injury as well as intrinsic factors, that is, skeletal factors such as bone strength, measured as density. The association between male sex and prevalent fracture has been reported in other contexts [1], and may relate to more risk-prone higher impact physical activities pursued by males, such as sport activities [30].

Fracture risk has been poorly studied in children with HIV. While there was no difference in fracture prevalence by HIV status, LS-BMAD appeared to be associated with prevalent fracture in CLWH, but not among uninfected children. Low bone density is a well recognized complication of HIV disease, with a 2.5 times higher odds of osteoporosis and/or osteopenia in adults living with HIV than in HIV-negative peers, including among individuals taking ART [31]. We and others have reported how children with perinatally acquired HIV also have lower bone density for age and sex, compared to HIV-uninfected peers [8,9]. In our study, we used size-adjusted bone density as CLWH often have growth delay, and areal bone density as measured by DXA can underestimate bone density in small children [18].

Growth delay, persistent immune activation and chronic inflammation driven by HIV infection and exposure to certain ART drugs can all affect bone accrual during critical periods of bone development in CLWH. Adolescence is a critical period for bone mass accrual; after cessation of linear growth, consolidation of mineral continues until peak bone mass (PBM) is achieved in early adulthood. Hence, disturbances in growth and bone accrual due to HIV infection have implications for achievement of PBM. Importantly, low PBM predicts *adult* osteoporotic fracture risk; a 10% reduction in PBM doubles fracture risk in adulthood [32]. Attention to potential fracture risk is therefore warranted in children with HIV reaching adulthood.

Physical activity is thought to have both beneficial and detrimental effects on fracture risk in childhood [1]; on the one hand, skeletal loading providing osteogenic stimulation is beneficial to bone mass [33], whilst injuries from vigorous physical activity can increase fracture risk irrespective of bone mass [24], with high-impact sports further increasing fracture risk [30]. The observed higher fracture prevalence in children without HIV is consistent with the higher levels of physical activity, some of which is expected to be vigorous and/or high impact; although, after adjusting for age and sex, physical activity was not associated with prevalent fracture in our analyses.

HIV-related factors such as CD4^+^ cell count, viral load and current tenofovir use were not associated with prevalent fractures in CLWH. However, these factors relate to the current point in time while the outcome measures events that may have occurred in the past. Furthermore, the study may have been underpowered to detect associations. In addition to HIV-specific risks, other traditional risk factors for low bone density and fracture include poor nutrition, low physical activity, inadequate dietary calcium intake and vitamin D deficiency. We note that in our study physical activity levels were lower in CLWH, but overall low dietary calcium and vitamin D intakes, as well as physical activity levels, were not associated with prevalent fractures.

We did observe differences in self-reported fracture management by HIV status, despite most fractures being confirmed within a health facility by X-ray, and all cases seeking medical attention for their fracture. CLWH were less likely to receive inpatient fracture care, despite no differences in fracture type by HIV status. This inequality may reflect lack of ability to pay for treatment, as suggested by the substantial differences by HIV status observed in socioeconomic status and warrant further investigation.

Disability was common, particularly in CLWH, in whom just over half reported a disability and/or functional impairment, mostly constituting sensory, memory and/or speech difficulties, as well as 8.3% having difficulty walking and/or running. The analyses were underpowered to detect associations between prevalent fracture and difficulty walking and/or running. The observed levels of self-reported disability were higher than previously reported in a similar Zimbabwean population, in whom 37.6% of 6–16 years old CLWH self-reported a disability, and 2.5% specifically difficulty walking [34]. Even at these previously reported levels, strong associations with impaired educational achievement and social functioning have been reported [34], highlighting the need for relevant support services within paediatric HIV programmes.

Study strengths include a broadly representative HIV-uninfected comparison group, recruited from the same catchment area as CLWH, and collection of detailed data on risk factors such as physical activity, dietary calcium and vitamin D intake using validated tools adapted for the local context. Recommended size-adjustment methods were used for DXA data, which were particularly important given height differences (levels of stunting) in the population studied [19,21]. We acknowledge several limitations: data are cross-sectional and therefore causality cannot be inferred. Fractures were self-reported so subject to recall bias. Given potential recall bias, the timing of fractures was not collected, hence it was not possible to calculate retrospective fracture incidence. Recall bias may be exacerbated among CLWH who are likely to have more experience of medical care. Lack of data on fracture mechanism prevented analysis by trauma level; we also lacked data on prenatal/perinatal HIV exposure in the children without HIV. The study was likely underpowered to detect a difference in fracture prevalence by HIV status, nor to determine an association between fracture and disability. As local or African reference population data are not available, *Z*-scores for TBLH-BMC^LBM^ and LS-BMAD were generated using data from a paediatric reference population in the UK, using the same manufacturer and software version to derive *Z*-scores (as recommended by international guidelines [19,21]), who may not be comparable to the children studied in Zimbabwe.

In conclusion, prevalent fractures appear to be associated with low size-adjusted bone density in CLWH. Research is needed to understand barriers to accessing fracture care. Continuing surveillance for fractures is warranted as the current cohorts of CLWH enter adulthood as are strategies to reduce the risk of developing future fractures.

## Acknowledgements

The authors thank Dr Nicola Crabtree for her advice regarding calculation of TBLH-BMC^LBM^ and LS-BMAD *Z*-scores.

Author's contributions: R.R., R.A.F., and C.L.G. conceived the study. R.A.F., C.L.G., R.R. and A.M.R. designed the study protocol. R.R. was responsible for data management. C.M.K. contributed to data collection. A.M.R., C.L.G., V.S. and R.R. conducted data analyses. R.R. wrote the first draft. R.R. and V.S. had full access to all the study data and had final responsibility for the decision to submit for publication. All authors contributed to the report and approved the final submitted text.

Author's information: R.R. (206764/Z/17/Z) and R.A.F. (206316/Z/17/Z) are funded by the Wellcome Trust. C.M.K. is funded by a National Institute of Health (NIH) Fogarty Trent Fellowship. A.M.R. and V.S. are partially supported by the UK Medical Research Council (MRC) and the UK Department for International Development (DFID) under the MRC/DFID Concordat agreement which is also part of the EDCTP2 programme supported by the European Union Grant Ref: MR/R010161/1.

### Conflicts of interest

There are no conflicts of interest.

## Supplementary Material

Supplemental Digital Content
