# Supplementary material for: Fracture prevalence and its association with bone density among children living with HIV in Zimbabwe
Source: AIDS. 2023 Feb 7;37(5):759–67. doi: 10.1097/QAD.0000000000003477 (PMC9994799; doi:10.1097/QAD.0000000000003477)
Supplement: Supplemental Digital Content [file aids-37-759-s001.docx]

**Supplementary Table 1: HIV specific risk factors for reporting a prevalent fracture**

| **Risk factor** | **Prevalent fracture (n=14)**  **N (%)** | **No prevalent fracture (n=289)**  **N (%)** | **Age/sex adjusted OR (95% CI)** | **p-value** |
| --- | --- | --- | --- | --- |
| Age at HIV diagnosis |  |  |  |  |
| <4 years | 9 (5.0) | 172 (95.0) | Ref | 0.736 |
| ≥4 years | 5 (4.1) | 117 (95.9) | -0.18 (-1.25, 0.88)^a^ |  |
| Missing | 0 (0.0) | 0 (0.0) |  |  |
| Age at ART initiation |  |  |  |  |
| <2 years | 3 (3.5) | 82 (96.5) | Ref | 0.869 |
| 2-3.9 years | 4 (5.5) | 69 (94.5) | 0.39 (-1.08, 1.85) ^a^ |  |
| 4-6 years | 7 (4.8) | 138 (95.2) | 0.29 (-1.04, 1.61) ^a^ |  |
| Missing | 0 (0.0) | 0 (0.0) |  |  |
| ART duration years |  |  |  |  |
| 2-5.9 years | 2 (2.9) | 66 (97.1) | Ref | 0.352 |
| 6-10 years | 7 (4.0) | 169 (96.0) | 0.29 (-1.26, 1.84) |  |
| >10 years | 5 (8.5) | 54 (91.5) | 1.03 (-0.60, 2.67) |  |
| Missing | 0 (0.0) | 0 (0.0) |  |  |
| % life on ART |  |  |  |  |
| <50% | 1 (1.3) | 76 (98.7) | Ref | 0.125 |
| 50-100% | 13 (5.8) | 213 (94.3) | 1.59 (-0.44, 3.63) |  |
| Missing | 0 (0.0) | 0 (0.0) |  |  |
| Current tenofovir use |  |  |  |  |
| Yes | 3 (2.9) | 99 (97.1) | -0.81 (-2.12, 0.49) | 0.221 |
| No | 11 (5.5) | 190 (94.5) | Ref |  |
| Viral load |  |  |  |  |
| <1,000 copies/ml | 8 (3.8) | 204 (96.2) | Ref | 0.587 |
| ≥1,000 copies/ml | 3 (5.4) | 53 (94.6) | 0.36 (-0.94, 1.67) |  |
| Missing | 3 (8.6) | 32 (91.4) |  |  |
| CD4 count |  |  |  |  |
| <500 cells/µL | 4 (6.9) | 54 (93.1) | Ref | 0.496 |
| ≥500 cells/µL | 10 (4.4) | 220 (95.6) | -0.40 (-1.54, 0.75) |  |
| Missing | 0 (0.0) | 14 (93.3) |  |  |
| Abbreviations: OR, odds ratio; CI, confidence interval   1. OR adjusted for sex only | | | | |

**Supplementary Figure 1. Fracture management by HIV status**
